# Assessing Health Implications of the Potential Radiation Exposure in the Community During Pregnancy: A Case Study

**DOI:** 10.7759/cureus.1770

**Published:** 2017-10-12

**Authors:** Jordan Wong, Haley Clark, Robert Corns, Scott Tyldesley

**Affiliations:** 1 Department of Radiation Oncology, BC Cancer Agency, Vancouver Centre; 2 Department of Medical Physics, BC Cancer Agency, Fraser Valley Centre

**Keywords:** radiation exposure, fetal radiation, radiation toxicity, radiation safety

## Abstract

The unintentional radiation exposure can have significant implications. We present a case of a 30-year-old pregnant female who was exposed to a potentially radioactive rock for over a one week period during her 13^th^ week of pregnancy. After an arduous process of obtaining activity measurements, the most conservative estimate of dose, the female was exposed to, was found to fall within the permissible limits. We briefly describe the literature on fetal radiation toxicity levels and discuss logistical issues faced in managing such cases.

## Introduction

The general public is constantly exposed to the radiation from both natural and human-made sources. Occasionally, the individuals become exposed to a radiation source unintentionally and are then concerned with the implications on their health. The radiation oncologists are the most suitable physicians to provide counseling, given their expertise pertaining to the radiation and their direct experience with the radiation exposure. The radiation safety officers (RSOs), who are typically trained as health or medical physicists and are capable of addressing radiation-related questions from the community. Here we discuss a case where a potentially radioactive source was brought into a home resulting in potential exposure and the perception of exposure to the household members including a pregnant female. We briefly describe the literature on radiation exposure limits and radiation toxicity in pregnancy and discuss the logistical issues faced in managing this case. Informed consent statement was obtained for this study.

## Case presentation

A 30-year-old female, 13 weeks pregnant, and her husband acquired a fluorite ore sample. After seven days with this sample on display on their kitchen counter, it was brought to their attention that the rock originated from a mining site in the United States rich in natural uranium deposits and likely contained radioactive substances. This disclosure generated significant distress and anxiety for the couple. In order to attempt to quantify their absorbed dosages and the risk to the fetus, they reached out to various organizations, including the Provincial Centre for Disease Control, Drug and Poisons Centre and the Environmental Health services in British Columbia. The female also consulted her family doctor who discussed a range of possible options including private testing of the ore sample (the results of which would take few weeks to arrive) or preemptive abortion of the fetus.

A local environmental health officer referred her to an RSO, who then brought the case to the attention of the British Columbia (BC) Cancer Agency administration and the Department of Radiation Oncology. After discussion, it was agreed that the dose rate measurements from the ore sample were required in order to make specific recommendations. It was also apparent that the matter should be dealt with in a timely fashion given the females' pregnancy.

Logistical difficulties were faced trying to obtain measurements, given that the role of the RSO does not encompass such unique circumstances, such as specifically attending the home of a person in the community to perform the required measurements. Ultimately, the police and the fire department’s hazardous materials (HAZMAT) unit were involved and measured the specific activity of the sample. Throughout the report, all dosimetric quantities have been converted from gray (Gy) to sievert (Sv) assuming purely photon and electron radioactive emissions. The activity was assessed using a Geiger meter and found the dose to be equivalent to the rate of 0.19 to 0.20 μSv/h which would result at “point-blank” range. It was not made clear if this measurement excluded natural background radiation, but the HAZMAT response mentioned that the reading was comparable to the background dose rate. No wipe tests were performed and the rock was confiscated. The female's exposure depends on the proximity to the source and the amount of time spent in its presence; for risk management purposes, a worst-case scenario was employed. If she were to have carried the sample in her pocket for 24 hours for all seven days, her cumulative exposure would result in an equivalent dose of almost 34 μSv. This radiation dose is significantly lower than the yearly permissible limit to the general public (1 mSv), and an even smaller fraction of the permissible limit for the pregnant nuclear energy workers (4 mSv). Furthermore, while a developing fetus is susceptible to radiation at 13 weeks post postconception, the risks of adverse effects are thought to be small and possibly nil under 100 mSv for photon radiation [1]. A radiation oncologist and the RSO met with the couple in consultation to reassure them that neither they nor the fetus had significantly increased their risk of medical problems from their exposure to the fluorite ore sample.

## Discussion

Radiation exposure limits

**Figure 1:**
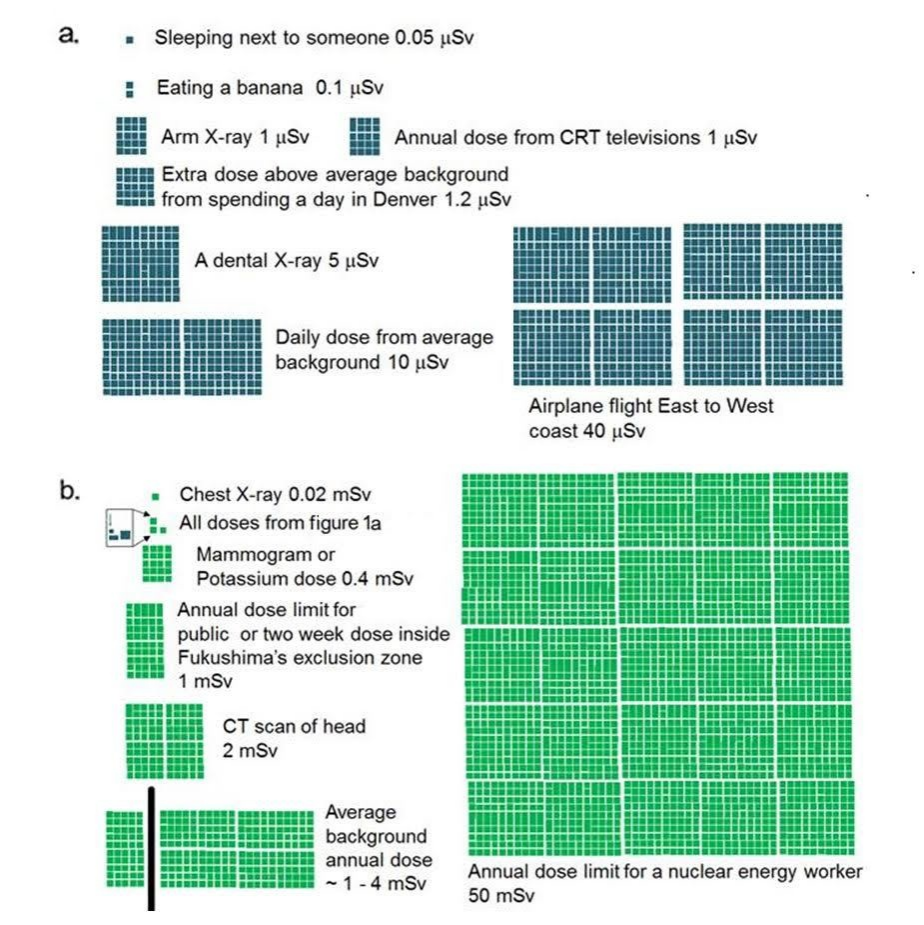
Approximate radiation doses of typical activities. The total dose of Figure 1a is represented in Figure 1b as three squares. The females' maximum dose would approximately equal two squares in Figure 1b. Adapted from XKCD [3] by Robert Corns.

According to the Canadian Nuclear Safety Commission’s (CNSC) Radiation Protection Regulations, the permissible absorbed radiation dose limit for a member of a general public is 1 mSv above the natural background per the calendar year [2]. While this document does not specify a different dose limit for pregnant individuals of the general public, it does specify a limit for pregnant nuclear energy workers, which is a cumulative effective dose of 4 mSv over the course of the pregnancy. To aid interpretation of the worst-case scenario, the couple was shown the following visual aids (Figure 1). This figure is based on an infographic developed for general education by a popular cartoonist in response to the public’s concern around the Fukushima accident in 2011 [3]. A dose of 34 μSv is similar to flying across Canada via a commercial airline or eating one banana a day for a whole year. The average annual effective dose from natural sources in Vancouver is 1.3 mSv, and the Canadian average is 1.8 mSv [4].

Radiation toxicity in pregnancy

The American Association of Physicists in Medicine (AAPM) has published a report reviewing fetal dose of radiotherapy with photon beams [1]. The International Commission on Radiological Protection also has reviewed the literature on the effects of prenatal irradiation dose and timing [5]. Both organizations agree that very little to no health concerns are expected at exposures resulting in doses < 50 mSv. The AAPM report summarizes the relative magnitude of the risks of certain health effects following irradiation depending on timing (Table 1) [1]. The absolute magnitude of these risks being quite small and possibly nil for absorbed doses lower than 100 mSv (Table 1).

**Table 1:** The estimated relative magnitude of risks associated with irradiation during fetal development periods. Absolute magnitude is felt to be small and possibly nil for absorbed doses < 100 mSv. The accuracy of estimates improves at higher doses. Adapted from The American Association of Physicists in Medicine (AAPM) [1].

Postconception Time (weeks)	0 to 1	2 to 7	8 to 15	16 to 25	>25
Lethality	High	Low	Low	None	None
Gross malformations	None	High	Low	Low	None
Growth retardation	None	High	Moderate	Low	Low
Mental retardation	None	None	High	Low	None
Malignant disease	None	Low	Low	Low	Low

Spontaneous abortions, congenital defects, and childhood cancers occur at varying frequencies in the general population. Table 2 compares the effects of 10 mSv dose in comparison to the natural prevalence of some of these issues [6]. The unit 10 mSv is 10 times the permissible limit to the general public and would itself be equivalent to a routine diagnostic computed tomography (CT) scan of the abdomen and pelvis. Table 2 also includes non-radiation exposures for additional perspective.

**Table 2:** The fetal effects of 10 mSv radiation dose and non-radiation exposures during pregnancy. Adapted from the UC Davis Safety Services [6].

Exposure	Effect	Number occurring from natural cause	Excess occurrences due to risk factor
Irradiationbefore birth	Cancer death	200 per thousand	0.2 per thousand
Irradiation four-seven weeks post gestation	Microcephaly	40 per thousand	5 per thousand
Irradiation eight-11 weeks post gestation	Microcephaly	40 per thousand	9 per thousand
Irradiation eight-15 weeks post gestation	Mental retardation	4 per thousand	4 per thousand
Work in high-risk occupations	Spontaneous abortion	200 per thousand	90 per thousand
Two-four drinks of alcohol per day	Fetal alcohol syndrome	1-2 per thousand	100 per thousand
Smoking less than one pack per day	Perinatal infant death	23 per thousand	5 per thousand

Expanding role of the radiation safety officers

In Canada, an RSO must be available 24 hours a day, seven days a week for any radiation-related issues or incidents at the Canadian Nuclear Safety Commission (CNSC) licensed facility. Their primary role is to control radiation sources, ensure they are being operated safely and in a manner consistent with the terms of the CNSC license, and to ensure the safety of any person who enters the facility. The radiation safety officers are themselves licensed by the CNSC to perform the assessment of radioactive substances and dose estimation. In particular, they are required to be able to assess dose in incidents involving ionizing radiation such as in this case. However, such cases are rare and during this case, there were institutional uncertainties about whether the RSO should be involved. The liability of the RSO in such a situation was unclear. While initial dose estimates were provided by the RSO, the liability issues forced measurements to ultimately be performed by a HAZMAT team. There was undoubtedly perceived situational escalation when a HAZMAT team retrieved the sample and surveyed the couple’s dwelling. While this process of community measurement of potential radiation hazard is presently necessary, the approach may have added undue anxiety and distress to an already stressful situation for the couple. Furthermore, even if the sample was found to be completely free of contaminants, the situation could have other ramifications; for example, being ostracized by neighbors or being evicted from their residence. We propose that the specific role of the RSO should be expanded to include consultation with members of the public who are neither staff nor patients with a licensed facility, but to also who are in need of RSO services (including community measurement of activity with such samples, when the low activity level is anticipated).

Response by the primary physician

In this case, the primary physician presented a range of choices to the couple that was bridled by two unknowns: the effect of radiation on the developing embryo, and the specific activity of the sample. Given the limitations inherent in the advice, the couple themselves sought the opinion of a professional capable of providing more informed advice pertaining to the radiation. The first option, to have the sample privately tested, was problematic given the long turnaround time expected. Fetal viability begins around 20 weeks post-conception; induced abortions performed beyond 21 weeks gestation are potentially dangerous and may be considered ethically grey procedures by some physicians [7-8]. The couple wished to avoid termination if at all possible, and significant emotional and ethical implications would likely have arisen had abortion been pursued. In hindsight, a preemptive abortion was not warranted in this case. In response to their proposed options, the couple acquired a dose rate meter and attempted to measure the sample and estimate accumulated dose themselves, but did not know how to interpret this information. Ideally, the family doctor would have been aware of the Centre for Disease Control (which offered a radiation estimation service that recently became defunct), the fire department capabilities, or (preferably) a health network specialist such as a radiation oncologist or RSO. The consultation with any such agencies before presenting options would have reduced distress.

The measurement was necessary

In this case, it was difficult to obtain accurate sample measurements. The measurements are necessary to make a specific recommendation owing to the complex nature of radioactivity and uncertainty about the chemical composition. It is not possible to accurately estimate radioactivity for a given sample without measurement, in this case, the uranium mining site was able to provide an upper limit estimate of the radioactive isotope concentration, but the dispersion of the materials within the sample, the composition of the non-radioactive materials, and geometry of the sample, amongst other factors, makes precise theoretical dose estimation difficult. The RSOs operating at CNSC-licensed facilities are required to be equipped with calibrated instruments capable of precise and accurate dose measurements. An RSO performing the measurement directly or greater cooperation with the HAZMAT team (e.g., in which the HAZMAT team retrieves the sample and the RSO performs the measurement, or in which the RSO advises the HAZMAT team for a tailored measurement procedure) would have presented a less intrusive approach for the couple. A written report was requested but not available by the time of consultation with the female and her husband, but verbal confirmation of the activity was received and an appropriate measurement device appeared to have been used.

Role of the radiation oncologist

Other considerations including medical history, family history, and physical examination could impact radiation risk and the advice given. The physician involvement is, therefore, an important component of the assessment. The role of the radiation oncologist typically includes consultation and the treatment of cancer patients with radiation therapy. Occasionally benign diseases are also treated with the radiation therapy. Given the specific training they receive and the working relationships they must have with the RSOs, we argue that radiation oncologists are the physicians best suited to provide consultation for the patients with radiation exposure concerns and the radiation oncologists should view consultation services to people with such potential radiation exposures, outside of their health agencies to be within their scope of practice. As in this case, it is our opinion that such consultations should be attended jointly by a radiation oncologist and an RSO.

## Conclusions

Our report details a case where a pregnant female was potentially exposed to an unknown dose of radiation by an ornamental rock. The ensuing process for determining fetal health risk was not a routine procedure. The radiation exposure can have significant effects on fetal development and it is important that the risk is quantified as soon as possible so the patients can make informed decisions. Besides the direct radiation risks, there are psychological, societal, and ethical factors to consider. A collaboration with a radiation safety officer and the radiation oncologists are the best physicians to counsel on such issues.

While radiation issues in the community are rare, the RSOs are available on call 24 hours a day, seven days a week to provide advice in terms of the management and assessment of radiation-related risks. Awareness of this resource would allow potential issues to be addressed as soon as possible. We propose that RSOs should have a close working relationship with the HAZMAT team and also have the ability to perform the measurements of the radiation activity in their communities in situations where risk is anticipated to be low.
